# Predicting the Impact of Climate Change on the Distribution of North China Leopards (*Panthera pardus japonensis*) in Gansu Province Using MaxEnt Modeling

**DOI:** 10.3390/biology14020126

**Published:** 2025-01-26

**Authors:** Yongqiang Yang, Wenjie Gao, Yapeng Han, Tianlin Zhou

**Affiliations:** 1 Gansu Key Laboratory of Protection and Utilization for Biological Resources and Ecological Restoration, Qingyang 745000, China; hanyap25@163.com (Y.H.); ldzysw@163.com (T.Z.); 2 School of Agriculture and Bioengineering, Longdong University, Qingyang 745000, China; 3 Xinjiang Key Laboratory for Ecological Adaptation and Evolution of Extreme Environment Biology, College of Life Sciences, Xinjiang Agricultural University, Urumqi 830000, China; w2268576787@163.com

**Keywords:** climate change, North China leopard, potential habitat, MaxEnt modeling, nature reserve

## Abstract

Climate change has a profound impact on the phenology and growth of vegetation, which in turn influences the distribution and behavior of animal communities, including prey species. This dynamic shift significantly affects predator survival and activities. This study utilizes the MaxEnt model to explore how climate change impacts the distribution of the North China leopard (*Panthera pardus japonensis*) in the Ziwuling region of Gansu Province, China. As an endemic subspecies and apex predator, the North China leopard is vital for maintaining the structure and function of local ecosystems. Unfortunately, its population faces several threats, including habitat change, interspecies competition, and human encroachment, all of which are compounded by the ongoing effects of climate change. To assess the requirement and quality of habitat for this species, we conducted a population survey in the Ziwuling area from May 2020 to June 2022, utilizing 240 infrared cameras, which identified 46 active leopard sites. Using the MaxEnt model, we simulated habitat suitability and future distribution under different climate change scenarios based on nine environmental variables. Our results indicate that the population distribution of North China leopards is primarily influenced by the mean diurnal range (Bio2), with additional sensitivity to isothermal conditions (Bio3), temperature seasonality (Bio4), maximum temperature of the warmest month (Bio5), and annual temperature range (Bio7). We also evaluated habitat suitability across three socioeconomic pathways (SSP126, SSP245, and SSP585) for three time intervals: the 2050s (2041–2060), the 2070s (2061–2080), and the 2090s (2081–2100). The findings suggest a significant decline in high-suitability habitat for North China leopards, while areas of medium and low suitability are projected to increase. Understanding these distributional changes in North China leopards will enhance our comprehension of the region’s biogeography and inform conservation strategies aimed at mitigating the impacts of climate change.

## Simple Summary

The North China leopard (*Panthera pardus japonensis*), an endemic subspecies and apex predator, plays a crucial role in maintaining local ecosystem structure and function. This study employs the MaxEnt model to assess the impact of climate change on the distribution of North China leopards in the Ziwuling region of Gansu Province, China. Our results indicate that the population distribution is primarily influenced by the mean diurnal range (Bio2), with additional sensitivity to isothermality (Bio3), temperature seasonality (Bio4), maximum temperature of the warmest month (Bio5), and annual temperature range (Bio7). Currently, the areas of high-, medium-, and low-suitability habitats measure 497.92 km^2^, 379.86 km^2^, and 406.25 km^2^, respectively. Evaluating habitat suitability across three socioeconomic pathways (SSP126, SSP245, and SSP585) at three time intervals (2050s, 2070s, and 2090s), we project a significant decline in high-suitability habitats for North China leopards, with increases in medium- and low-suitability areas. These findings underscore the need for ongoing monitoring and research to understand the population dynamics and threats faced by North China leopards, facilitating the development of scientifically robust conservation plans.

## 1. Introduction

Ecologists have long emphasized the correlation between species distribution and the environment, pinpointing the pivotal influence of climatic factors on species distribution [1]. Climate change impacts can be observed at the level of individual species and entire communities [2]. As global warming progresses, many animals and plants are slowly shifting towards higher latitudes or elevations [3,4]. Between 2011 and 2020, the global temperature rose by 1.1 °C, with a further predicted increase between 3.3 °C and 5.7 °C by the end of the 21st century [5]. In China, the average temperature of the country’s land area increased by 0.9 °C to 1.5 °C from 1909 to 2011, with a projected further increase of 1.3 °C to 5.0 °C by the end of the 21st century [6]. Observational data have indicated that climate change has a significant impact on the structure and function of ecosystems, the composition and distribution range of biological communities, and biological phenology, in addition to causing increases in habitat fragmentation, resulting in an acceleration of species extinction [7,8,9,10]. To adapt to climate change, many species are gradually moving to climates more suitable for their survival, leading to changes in species distribution [11,12]. According to the results of Thomas et al. [2], if climate change continues unchecked, 37% of global species is expected to become extinct by the end of 2050, which is a serious issue for all species, as well as the functioning of ecosystems and services [13]. *Panthera pardus* is a large carnivore. Compared with other large carnivores, it has a better environmental adaptability and can adapt well to a variety of habitats, forests, grasslands, and farmland [14]. Leopards possess a broad diet and occupy a diverse ecological niche, which enhances their adaptability to environments altered by human activities [15,16,17,18]. As apex predators, large carnivores play a critical role within ecosystems, occupying higher trophic levels that significantly influence prey populations and their distribution [18,19]. Moreover, their presence impacts various biological and abiotic components at multiple trophic levels through cascading nutrient interactions [19,20]. Consequently, apex predators are essential for maintaining ecosystem integrity and stability [21,22]. In an ecosystem, reductions in the population of large carnivores usually causes a series of serious ecological problems such as a decline in overall biomass, reductions in primary productivity, and the loss of key ecological functions in the ecosystem [19,20,23]. Moreover, the ecological niche over large carnivores cannot be replaced by small and medium-sized carnivores [20].

The North China leopard (*Panthera pardus japonesis*) is a subspecies of the leopard, a large feline that was formerly widely distributed in most areas of Northern China. Currently, the North China leopard is facing a series of threats, such as fragmentation and declining habitat quality, small and isolated populations, and human–wildlife conflicts [14,24]. Due to hunting and habitat destruction by humans, the population and distribution range of the North China leopard has decreased sharply, even becoming extinct in many areas of China and being listed as a national-level key protected wild animal [25]. Thus, predicting the potential distribution pattern of species under future climate change scenarios is important to effectively monitor species, conduct appropriate habitat management, and protect biodiversity.

With the development of the Internet and GIS science, niche models are often used in ecology and biogeography to study the impact of climate change on species distribution patterns [26]. Moreover, prediction models can be used to forecast changes in the distribution and abundance of species [13]. Species distribution models (SDMs) involve linking geocoded information on species distributions to environmental conditions and selecting appropriate algorithms to predict the future distribution range of a species. These models are important tools in ecology, biogeography, and conservation biology [1,27]. There are several commonly used niche models in ecology, including maximum entropy (MaxEnt) models [28], bioclimatic modeling (BIOCLIM) [1], genetic algorithm for rule-set prediction (GARP), and domain environmental envelope (DOMAIN) models [29,30]. These models can predict potential habitat areas independently. The MaxEnt model is commonly utilized for analyzing the potential distribution of species in response to future climate change scenarios. This model predicts species distribution in a specific area by examining the relationship between the current distribution of species and environmental factors [31]. Its predictions are easily understandable and highly accurate, making it one of the preferred methods for species distribution modeling [28,32,33].

In recent years, the strengthening of ecological and wildlife protection measures has led to a gradual increase in the population of North China leopards, with the species reappearing after a period of presumed extinction. Relevant studies on North China leopards have begun to emerge, primarily focusing on their distribution status [34,35], population size [36,37,38], their relationship with prey [39,40], and habitat selection, in addtion to protection suggestions [27,41]. However, there is a notable scarcity of research regarding the habitat of North China leopards, and no literature reports exist on their habitat suitability in the Ziwuling area of Gansu Province. This study aims to analyze the habitat suitability of the North China leopard population in Ziwuling, Gansu Province, utilizing infrared camera monitoring data. The goal is to enhance the protection of this flagship species and provide reasonable management suggestions for wildlife protection in the Ziwuling reserve.

## 2. Materials and Methods

### 2.1. Study Area

**Figure 1:**
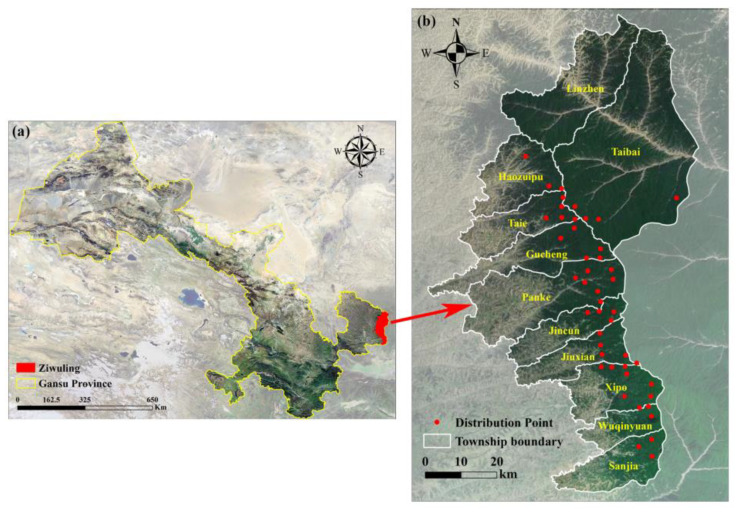
Map showing Gansu Province, China (**a**), and the study area—Ziwuling Forest Area (**b**).

Ziwuling is located in the hinterland of the Loess Plateau, between the Jing River and the Luo River, and is the boundary line between the Shaanxi and Gansu Provinces. The mountain trend is north–south, and because it is parallel to the prime meridian, it has been called “Ziwu Ridge” historically. It is located at N: 107°30′–109°40′, E: 33°50′–36°50′ (Figure 1), with an altitude of approximately 1100~1900 m [42]. It stretches 413 km from north to south and is 60–80 km wide from east to west. The total area reaches 23,000 km^2^, including 11,000 km^2^ in Gansu and 12,000 km^2^ in Shaanxi. The Ziwuling forest area is the largest and most representative natural secondary forest in the middle of the Loess Plateau [43]. The forest cover is as high as 80%; trees are lush, and the animal and plant resources are abundant. Known as the Green Great Wall, the Ziwuling area is an important natural reservoir, of natural medicine and biodiversity, and it is an important ecological barrier between the Longdong Loess Plateau and the Shaanbei Loess Plateau. It plays an extremely important ecological function in regulating climate, conserving water sources, maintaining soil and water cycles, reducing pollution, and preserving biodiversity. It is one of the 35 priority areas for biodiversity protection in China [44].

### 2.2. Species Data

In 2020, the core area of the Ziwuling Nature Reserve in Gansu Province was gridded into 240 sample areas, defined by a grid of 3.6 km × 3.6 km squares, utilizing geographic information system (GIS) technology (ArcGIS 10.8, Esri, Redlands, CA, USA). An infrared camera was installed in each sample area, resulting in a total of 240 infrared cameras. The cameras were deployed from May 2020 to July 2022, and the specific model used was the Ereagle-E3H (Beijing Prestar Tech. Co., Ltd., Beijing, China). The camera locations were typically selected near ridges, hillsides, valley bottoms, forest paths, animal trails, or water sources. The installation height of each camera was determined by the environmental conditions affecting the lens’s field of view, with a general installation height of approximately 40–50 cm and a distance of 3–5 m from potential animal routes. Ground vegetation and other obstructions in the camera’s field of view were cleared, and no bait was placed near any of the camera traps. To minimize false triggers, the camera sensitivity was set to medium [38,45]. From May 2020 to July 2022, each camera trap was operated for a duration of six months, after which its functionality was assessed. During this evaluation, the SD card and battery were replaced to ensure continuous operation, and any missing or damaged cameras in specific sample areas were reinstalled. In total, 240 cameras captured over 1.6 million images throughout the study. Among the 240 sample areas, North China leopards were photographed in a total of 46 areas (Figure 1).

### 2.3. Environmental Data

Environmental variables play a crucial role in elucidating the distribution of habitats from an ecological perspective, particularly in relation to the ecological niche of a species [46]. In this study, altitude and 19 bioclimatic variables in the current era (1970–2000) and in future eras of the 2050s (2041–2060), 2070s (2061–2080), and 2090s (2081–2100) were downloaded from WorldClim v2.1 (http://www.worldclim.org/, accessed on 2 September 2024). All environmental datasets were provided at a resolution of 30 arc-seconds and were converted to ASCII raster files, which are essential for use in MaxEnt. The future (2050s, 2070s, and 2090s) climatic variables were selected from the “HadGEM3-GC31-LL” climate model, which is one of the 49 climate model scenarios from the 2021 IPCC sixth assessment report (AR6). According to Meinshausen et al. [47], the shared socioeconomic pathways (SSPs) include five main SPPs (SSP 119, SSP 126, SSP 245, SSP 370, and SSP 585). Among these, we used three shared SSPs (SSP 126, SSP 245, and SSP 585) for three time steps: 2050s (2041–2060), 2070s (2061–2080), and 2090s (2081–2100) [48,49].

### 2.4. Variable Selections

Because of the multicollinearity among environmental variables, the predicted distribution is often overfitted. To mitigate this issue, 20 environmental variables were screened. The screening process involved two main steps: (1) the Correlation function in ENMTools 1.4 was utilized to calculate the correlations among the 20 environmental variables, applying a significance threshold of 0.80, and (2) MaxEnt 3.4.3 software (Columbia University, NY, USA) was employed to analyze species distribution data alongside 20 environmental variables, yielding preliminary percentage contributions and jackknife analysis results for each variable in the model. Subsequently, factors exhibiting a correlation coefficient greater than 0.80 and a lower contribution rate were removed, leading to the selection of the final environmental variables for model construction [50]. The environmental variables of the final selection model were Bio1, Bio2, Bio3, Bio4, Bio5, Bio6, Bio7, Bio11, and Ele (Table 1).

### 2.5. MaxEnt Model

*P. pardus japonensis* distribution point data (n = 46) and selected environment variables were imported into MaxEnt 3.4.4 software. The data were randomly divided: 75% of the sample data were used as training data and the test data comprised the remaining 25% and were used to validate the MaxEnt model [51,52]. The maximum number of iterations was set as 10,000, the model was repeated 10 times, and the predicted results were output in the “Logistic” format and the “ASC” file type. Other parameters were set to the default values [53]. The habitat suitability curves for each variable were calculated, and the contributions of each variable to the *P. pardus japonensis* habitat model were calculated using the software’s built-in jackknife test with ten repetitions [54]. The results of the built-in jackknife test indicate the extent of gain derived from each variable in isolation, as well as from the collective influence of all variables. A higher gain value associated with an individual variable suggests that it contains more information or contributes significantly to the distribution of species habitats [50,55].

Following the completion of MaxEnt modeling, the receiver operating characteristic (ROC) curve was utilized to assess the model’s performance, with the area under the curve (AUC) serving as a metric for accuracy. A positive correlation was observed between the area under the curve and the predictive performance of the model [32,56]. In this study, the average AUC derived from ten calculation results is utilized as a criterion for evaluating model performance. Generally, the AUC should range between 0.5 and 1. An AUC value of 0.5 indicates that the model’s performance is equivalent to random guessing. Values between 0.5 and 0.6 are considered unqualified, 0.6 to 0.7 are deemed poor, 0.7 to 0.8 are classified as average, 0.8 to 0.9 are regarded as good, and between 0.9 and 1.0 are classified as excellent [57].

After conducting the MaxEnt modeling analysis, the production results were imported into ArcGIS 10.8, where they were analyzed using a map of Ziwuling as the underlying layer. The grades of suitable habitats were reclassified using the equidistance classification method in Spatial Analyst Tools. The threshold was determined based on the specific research object and its requirements. Previous studies have indicated that a larger threshold should be selected for model predictions when focusing on invasive or potentially harmful species. This approach facilitates the allocation of limited resources to regions where they are most needed. Conversely, a smaller threshold should be adopted to protect endangered species when they are the subject of research [58,59,60]. Referring to the methodology outlined in the Fourth Assessment Report of the Intergovernmental Panel on Climate Change (IPCC) for assessing likelihood, a relatively small threshold of 0.2 was selected as the criterion for appropriate regional classification. The suitability scaling comprises four grades: unsuitable habitats (*p* < 0.2), minimally suitable habitats (0.2 ≤ *p* < 0.4), moderately suitable habitats (0.4 ≤ *p* < 0.6), and highly suitable habitats (*p* ≥ 0.6) [50].

## 3. Results

### 3.1. Model Performance and Variable Contributions

**Figure 2:**
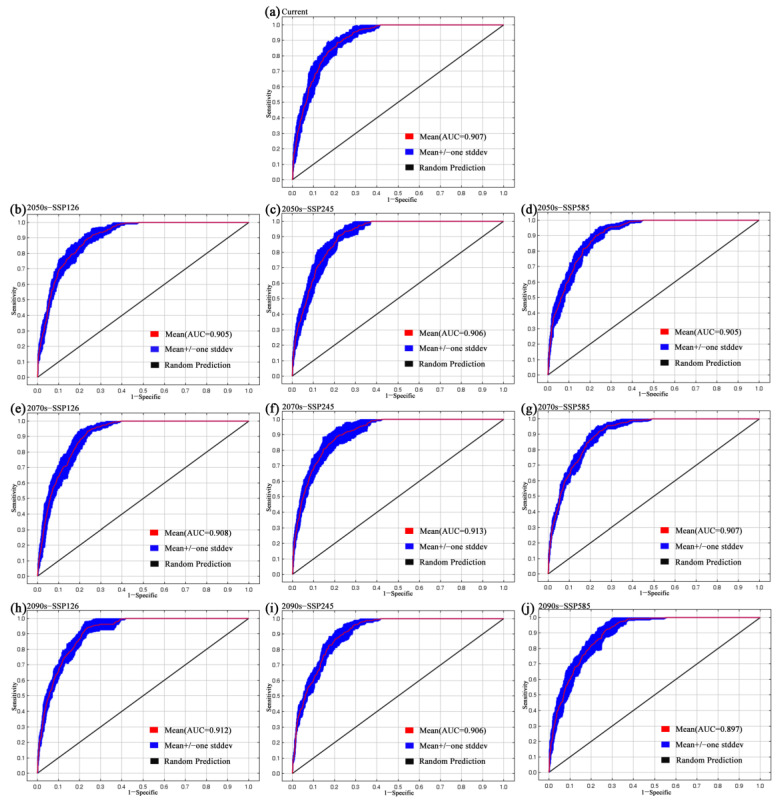
AUC values of *P. pardus japonensis* by MaxEnt model. (The red (training) line shows the “fit” of the model to the training data. The blue (testing) line indicates the fit of the model to the testing data and is the real test of the model’s predictive power.)

MaxEnt simulations for contemporary times and future scenarios (2050s, 2070s, and 2090s) were conducted using nine parameters, with the AUC results presented in Figure 2. The area under the receiver operating characteristic curve (AUC) values exceeded 0.89 for all models (Figure 2). These findings indicate that the MaxEnt model is highly reliable in predicting potential suitable areas for *P. pardus japonensis*.

Percent contribution (PC) and permutation importance (PI) serve as the primary indicators for assessing the significance of environmental variables. A higher index value signifies greater importance among these variables. The top five environmental variables ranked by percent contribution are as follows: precipitation of the mean diurnal range (bio2, 25.50%), isothermality (bio3, 20.90%), annual temperature range (bio7, 14.50%), mean temperature of the coldest quarter (bio11, 13.60%), and temperature seasonality (bio4, 10.40%), collectively accounting for 84.90% of the total contribution. In terms of permutation importance, the five leading environmental variables are isothermality (bio3, 29.80%), min temperature of coldest month (bio6, 22.10%), mean temperature of the coldest quarter (bio11, 14.70%), mean diurnal range (bio2, 8.70%), and temperature seasonality (bio4, 8.10%), which together account for 83.40% of the overall importance (Table 1).

**Figure 3:**
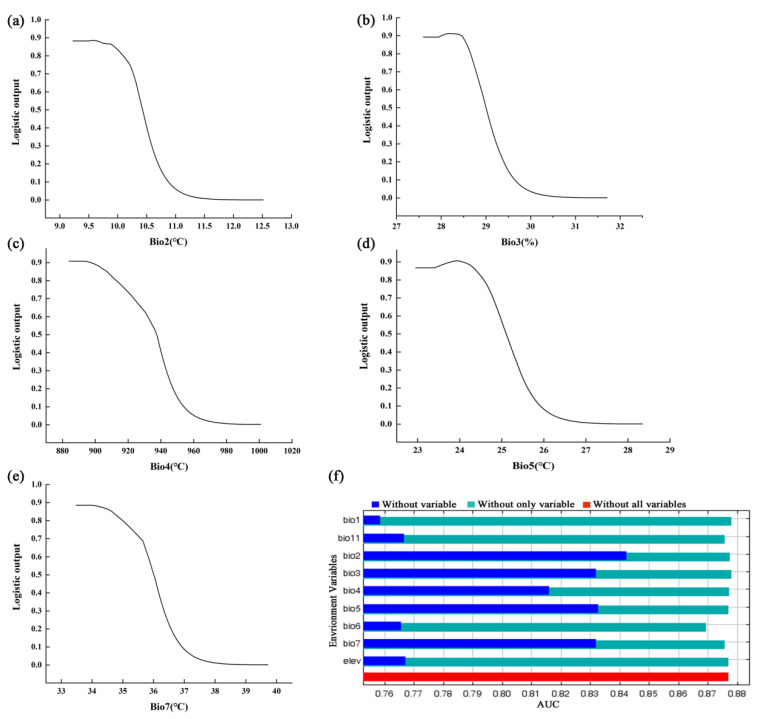
The response curves and jackknife test of environmental variables. (**a**–**e**) The response curves of the mean diurnal range, isothermality, temperature seasonality, max temperature of warmest month, and temperature annual range, respectively. (**f**) The contribution of each environmental factor to each scenario using the jackknife test on the AUC.

The contributions of the nine variables, as determined using the jackknife test, are presented in Figure 3, where five variables demonstrate gains greater than 0.8, specifically Bio2, Bio3, Bio4, Bio5, and Bio7. These results suggest that these variables may provide more valuable information compared to the others. Bio1 and elevation exhibited moderate gains when assessed individually, whereas Bio6 and Bio11 showed lower gains.

### 3.2. Current and Future Potential Suitable Areas for P. pardus japonensis and Their Spatiotemporal Changes

**Figure 4:**
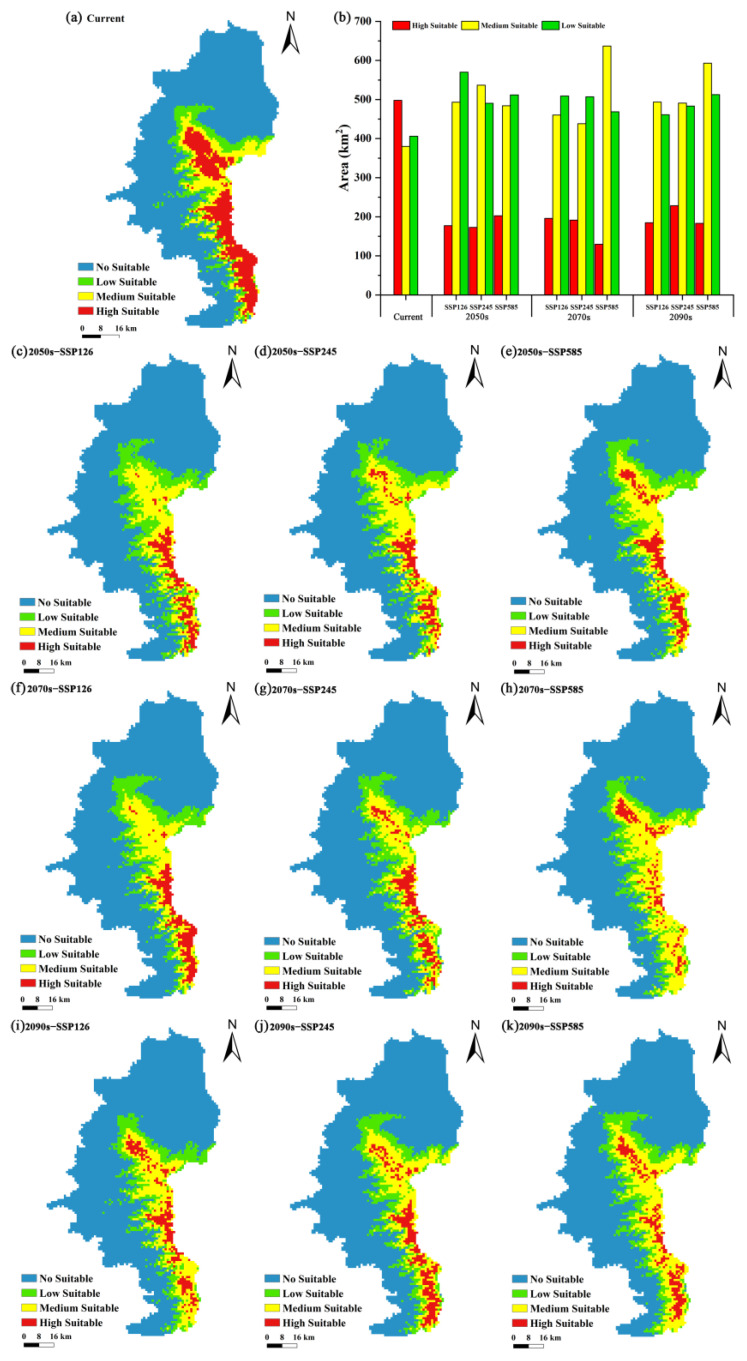
The predicted suitable habitat distributions of *P. pardus japonensis* in the Ziwuling provincial nature reserve in Gansu Province: (**a**) the current distribution; (**b**) statistical maps of different suitable areas for *P. pardus japonensis* in Ziwuling in different periods; (**c**–**e**) suitable habitats in the 2050s under the different SSPs; (**f**–**h**) suitable habitats in the 2070s under the different SSPs; (**i**–**k**) suitable habitats in the 2090s under the different SSPs.

Based on equal interval classification, the distribution of suitable habitats for *P. pardus japonensis* was reclassified into four categories: unsuitable habitats (*p* < 0.20), low-suitability habitats (0.20 < *p* < 0.40), medium-suitability habitats (0.40 < *p* < 0.60), and high-suitability habitats (*p* > 0.60). The *p* value represents the habitat suitability for the species as determined by the model. Furthermore, the distribution of potential and suitable habitats for *P. pardus japonensis* is illustrated in Figure 4. The future distribution range of the potential suitable habitats of *P. pardus japonensis* is generally the same, mainly concentrated in the core of the reserve area, at the border of Gansu and Shaanxi Provinces (Figure 4).

According to the MaxEnt simulation results, the contemporary potential total suitable area of *P. pardus japonensis* is 1284.03 km^2^, accounting for 20.76% of the study area. This habitat distribution is composed of a high-suitability area of 497.92 km^2^, accounting for 8.05% of the total; a medium-suitability area of 379.86 km^2^, accounting for 6.14%; and a low-suitability area of 406.25 km^2^, accounting for 6.57%. In the 2050s’ scenario, the proportion of the total suitable area for the North China leopard under the studied socioeconomic pathways (SSP126, SSP245, and SSP585) will be 20.05%, 19.40%, and 19.38%, respectively. In the 2070s, the proportions of the total suitable area of *P. pardus japonensis* under the studied socioeconomic pathways (SSP126, SSP245, and SSP585) will be 18.84%, 18.38%, and 19.97%, respectively. In the 2090s, the proportions of the total suitable area of *P. pardus japonensis* under the studied socioeconomic pathways (SSP126, SSP245, and SSP585) will be 18.42%, 19.44%, and 20.83%, respectively, which are lower than the contemporary proportion of 20.76%, except for the proportion in the 2090s under SSP585 (Table 2; Figure 4). In the 2050s, the proportion of suitable areas exhibited an overall downward trend under the socioeconomic pathways SSP126, SSP245, and SSP585. In contrast, in the 2070s and 2090s, the proportion of suitable areas shows an upward trend.

**Figure 5:**
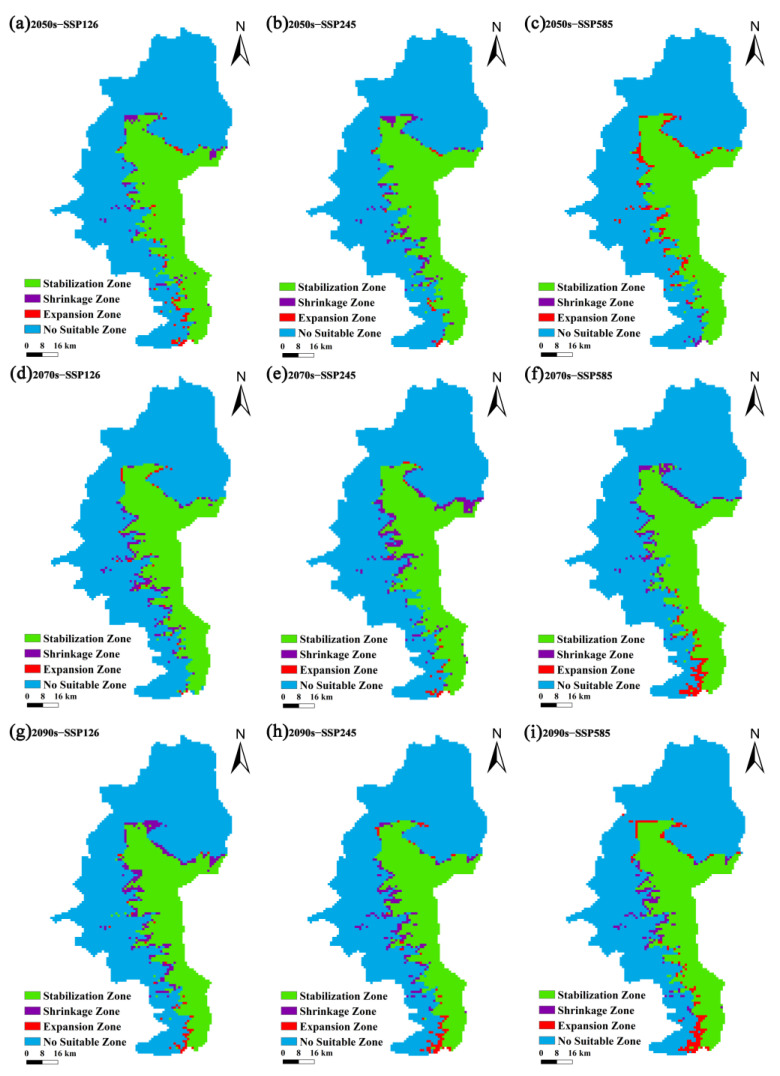
Spatial transformation pattern of suitable areas for *P. pardus japonensis* in different periods. (**a**–**c**) Greenhouse gas emission concentrations projected for the period 2041–2060, presented in lowest, middle, and highest scenarios; (**d**–**f**) greenhouse gas emission concentrations for the period 2061–2080, presented in lowest, middle, and highest scenarios; (**g**–**i**) greenhouse gas emission concentrations anticipated for the period 2081–2100, presented in lowest, middle, and highest scenarios. The increase in and loss of suitable areas is derived and compared to the current suitable area.

Compared to the current potential distribution range of *P. pardus japonensis*, the area of suitable habitats under SSP 126 is projected to shrink significantly. Specifically, the suitable areas are expected to decrease by 6.25 km^2^ and 9.03 km^2^ in the 2050s and 2070s, respectively, under this scenario. However, by the 2090s, the newly expanded suitable area is anticipated to surpass the contracted area, resulting in an increase of 38.89 km^2^, which represents 2.42% of the current area. Under SSP 245, the expansion of suitable habitats in the 2050s and 2090s is also expected to exceed the initial contraction, with increases of 53.47 km^2^ and 7.64 km^2^, respectively, accounting for 3.33% and 0.48% of the current area. In the 2070s, the suitable area is projected to decrease by 61.11 km^2^, representing 3.81% of the current area. According to SSP 585, the newly expanded potential suitable area in the 2050s and 2070s is expected to be larger than the contracted area, with increases of 17.36 km^2^ and 18.05 km^2^, respectively, corresponding to 1.08% and 1.12% of the current area. However, in the 2090s, the contracted area is anticipated to exceed the expanded area, leading to a decrease of 10.41 km^2^ (Table 3, Figure 5).

In summary, the results reveal a significant decline in high-suitability habitat for North China leopards, while areas classified as medium and low suitability are projected to increase (Figure 4). Based on these findings, we anticipate that the potential suitable habitat for *P. pardus japonensis* in the Ziwuling area will trend southwestward, whereas the potential suitable areas in the northern regions are expected to remain largely unchanged (Figure 5).

## 4. Discussion

### 4.1. Modeling Performance

Currently, among the reported species distribution models, the MaxEnt model has better stability and higher accuracy, and it exhibits less distortion in dealing with group temperature factors [28,32,61]. Variable selection has a remarkable effect on species distribution modeling [62]. Terrestrial ecosystems exhibit a high sensitivity to temperature changes induced by climate change, which can directly or indirectly influence the spatial distribution patterns of species and their associated ecological factors. This, in turn, alters the distribution areas, ranges, and population sizes of various species [63,64]. In recent years, climate change has significantly impacted the habitats of many species; for example, the rise in temperature has compelled certain species to contract their habitats and has even led to local extinctions [65,66]. Research indicates that around 1870, the average temperature in China increased rapidly, coinciding with a marked rise in habitat loss for leopards, thereby suggesting a clear correlation between temperature changes and the distribution of leopard habitats [67]. In this paper, temperature and altitude related to the characteristics of leopards are selected as the main climatic factors to make related predictions. A preliminary analysis of gain or permutation importance using MaxEnt provides a more objective tool for selecting variables by assessing their impact on the accuracy of the model. Based on this preliminary analysis, highly correlated variables have been screened and removed using ENMTools 1.4, thereby enhancing prediction accuracy. The model prediction results indicate that the AUC and TSS values for both current and future time periods (2050s, 2070s, and 2090s) are all greater than 0.85, demonstrating the high accuracy and discriminative capability of the predictions. Furthermore, the results from the MaxEnt model suggest that the potential suitable habitats for *P. pardus japonensis* are primarily located in the core area of Ziwuling, which aligns with our actual investigation findings.

### 4.2. Main Environmental Factors Affecting the Distribution of P. pardus japonensis

The response curves of environmental variables and their relationship with habitat suitability will provide more detailed insights into the habitat requirements of *P. pardus japonensis*, provided that the predictive statistical responses for distribution closely align with the species’ actual ecological responses. Consequently, these values could serve as a reference range for studies in population ecology and biology [46]. The results from the jackknife, area under the curve (AUC), percent contribution (PC), and permutation importance (PI) analyses indicate that the significance of variables such as mean diurnal range (Bio2), isothermality (Bio3), temperature seasonality (Bio4), max temperature of warmest month (Bio5), minimum temperature of the coldest month (Bio6), annual temperature range (Bio7), and mean temperature of the coldest quarter (Bio11) plays a crucial role in influencing the distribution of *P. pardus japonensis*. The impact of temperature fluctuations on leopards may manifest both directly and indirectly. Changes in air temperature directly affect vegetation phenology and growth, which in turn influence the distribution and behavioral patterns of prey and other animal communities. These dynamics can significantly impact the survival and activity of leopards as apex predators [67]. The results indicate that the optimal habitat for *P. pardus japonensis* is associated with a mean diurnal range of 9.23 to 10.43 °C, an isothermality of 27.60 to 29.00%, a temperature seasonality range of 884.00 to 937.42 °C, a max temperature of warmest month range of 22.95 to 25.10 °C, and an annual temperature range between 33.48 and 36.01 °C (Table 4).

### 4.3. Changes in Potential Suitable Areas

Compared with the current time period, under the three shared socioeconomic pathways in the future, the area of low-suitability habitat changed less than that of high-suitability habitat, and the area of potentially suitable habitats showed an overall downward trend. The change in air temperature may directly affect the birth rate, reproduction, the survival of female cubs and adults, and the health status of individual animals, which leads to the decrease in leopard habitat distribution [67]. Compared to the current conditions, under the shared socioeconomic pathways studied in this work (i.e., SSP126, SSP245, and SSP585), the areas of low- and medium-suitability habitats for *P. pardus japonensis* exhibited an increasing trend, while the area of highly-suitable habitat decreased by approximately 331.20 km^2^, 269.45 km^2^, and 314.59 km^2^. Habitat suitability reflects the availability of resources necessary for the survival and reproduction of wild animals within their home range, and high habitat suitability provides wildlife with sufficient resources [68]. With the decline in highly suitable areas (optimal habitats) and the increase in low- and medium/moderate-suitability ones (sub-optimal areas), leopards are expected to inhabit more of these less suitable environments. This shift may lead to increased human–wildlife conflict and a decreased growth rate of the leopard population in the future, which has significant implications for the management of this species.

### 4.4. Conservation Strategies for P. pardus japonensis

Global warming, the increase in extreme weather events, and anthropogenic alterations to mountains and rivers have significantly impacted the ecological balance of biological habitats [52]. In the Ziwuling region, the North China leopard is the largest carnivorous feline and occupies the apex position in the food chain. This species plays a crucial role in regulating prey populations and maintaining the overall balance of the ecosystem. Exploring the changes in the suitable distribution areas of the North China leopard under different climatic conditions in the future is of great significance for protecting this species and can also provide an important basis for the planning and development of nature reserves.

This study suggests the following measures for the conservation of *P. pardus japonensis*. First of all, continuous monitoring and research on the population of the North China leopards should be conducted to understand the dynamics of their population development and threatened status. Secondly, various media systems should be leveraged to promote knowledge, policies, and regulations related to the protection of the North China leopard and strengthen awareness of the threats to its survival. Thirdly, to develop a comprehensive and effective conservation strategy, future studies should assess the habitat distributions and population numbers of all major prey species of *P. pardus japonensis*. Only through this holistic approach can we accurately determine the suitable habitats for North China leopards and create informed and scientifically robust conservation plans.

## 5. Conclusions

In this study, the MaxEnt model for *P. pardus japonensis* yielded satisfactory results. These predictive outcomes provide significant insights for the development and conservation of *P. pardus japonensis*. Additionally, our findings contribute to the conservation of leopard diversity and enrich biogeographical research. The results indicate a decline in highly suitable habitats while predicting an increase in low- and medium-suitability habitats. Moreover, the distribution of *P. pardus japonensis* is predominantly influenced by environmental factors, such as the mean diurnal range (Bio2), isothermality (Bio3), temperature seasonality (Bio4), maximum temperature of the warmest month (Bio5), minimum temperature of the coldest month (Bio6), annual temperature range (Bio7), and mean temperature of the coldest quarter (Bio11). This suggests that the species is sensitive to temperature fluctuations, making it vulnerable to climate change. To mitigate the effects of climate change and protect the habitat of *P. pardus japonensis*, it is essential to adopt a low-carbon lifestyle in the future. Furthermore, improving reserve management and wildlife protection strategies will also be crucial.

## Figures and Tables

**Table 1:** Environmental variables and contribution rates (%) to *P. pardus japonensis*.

Code	Bioclimatic Variables	Unit	PC (%)	PI (%)
Bio1	Annual Mean Temperature	°C	2.40	5.40
Bio2	Mean Diurnal Range	°C	25.50	8.70
Bio3	Isothermality	%	20.90	29.80
Bio4	Temperature Seasonality	°C	10.40	8.10
Bio5	Max Temperature of Warmest Month	°C	7.00	6.60
Bio6	Min Temperature of Coldest Month	°C	5.40	22.10
Bio7	Temperature Annual Range	°C	14.50	3.60
Bio11	Mean Temperature of Coldest Quarter	°C	13.60	14.70
Ele	Elevation	m	0.50	0.90

**Table 2:** Statistics of suitable areas for *P. pardus japonensis* under different climate scenarios.

Circumstances	Low Suitability		Medium Suitability		High Suitability		All	
	Area (km^2^)	Percentage (%)	Area (km^2^)	Percentage (%)	Area (km^2^)	Percentage (%)	Area (km^2^)	Percentage (%)
Current	406.25	6.57	379.86	6.14	497.92	8.05	1284.03	20.76
2050s-SSP126	570.14	9.22	493.06	7.97	177.08	2.86	1240.28	20.05
2050s-SSP245	490.28	7.93	536.81	8.68	172.92	2.80	1200.01	19.40
2050s-SSP585	511.81	8.27	484.03	7.82	202.78	3.28	1198.62	19.38
2070s-SSP126	509.03	8.23	460.42	7.44	195.83	3.17	1165.28	18.84
2070s-SSP245	506.94	8.19	438.19	7.08	191.67	3.10	1136.8	18.38
2070s-SSP585	468.75	7.58	636.81	10.29	129.86	2.10	1235.42	19.97
2090s-SSP126	461.11	7.45	493.75	7.98	184.72	2.99	1139.58	18.42
2090s-SSP245	483.33	7.81	490.97	7.94	228.47	3.69	1202.77	19.44
2090s-SSP585	512.5	8.28	593.06	9.59	183.33	2.96	1288.89	20.83

Note: The area percentages are the ratios of the suitable areas of different grades to the total study area (6186.29 km^2^) under different climate scenarios.

**Table 3:** Spatial variations in suitable habitat for *P. pardus japonensis* in different periods.

Circumstances	Area (km^2^)			Rate of Change (%)		
	EZ	SZ	Change	EZ	SZ	Change
2050-126	53.47	59.72	6.25	3.33	3.72	0.39
2050-245	75.00	21.53	53.47	4.67	1.34	3.33
2050-585	88.19	70.83	17.36	5.50	4.41	1.08
2070-126	50.00	59.03	9.03	3.12	3.68	0.56
2070-245	38.89	100.00	61.11	2.42	6.23	3.81
2070-585	65.97	47.92	18.05	4.11	2.99	1.12
2090-126	68.75	29.86	38.89	4.28	1.86	2.42
2090-245	75.00	67.36	7.64	4.67	4.20	0.48
2090-585	66.67	77.08	10.41	4.15	4.80	0.65

Note: The rate of change is the percentage of the area of each period and the area of the contemporary suitable area. The current area of potential suitable habitat for *P. pardus japonensis* is 1284.03 km^2^. EZ: expansion zone; SZ: shrinkage zone.

**Table 4:** Suitable range and optimal value of each environmental variable for *P. pardus japonensis*.

Environmental Variables	Suitable Range	Optimal Value
Bio1: Annual Mean Temperature (°C)	6.87–8.16	7.70
Bio2: Mean Diurnal Range (°C)	9.23–10.43	9.57
Bio3: Isothermality (%)	27.60–29.00	28.45
Bio4: Temperature Seasonality (°C)	884.00–937.42	884
Bio5: Max Temperature of Warmest Month (°C)	22.95–25.10	23.94
Bio6: Min Temperature of Coldest Month (°C)	−11.1–−10.27	−10.46
Bio7: Temperature Annual Range (°C)	33.48–36.01	33.48
Bio11: Mean Temperature of Coldest Quarter (°C)	−4.17–−3.57	−3.82
Elevation (m)	1454.14–1723.06	1694.34

## Data Availability

The original contributions presented in this study are included in the article. Further inquiries can be directed to the corresponding author(s).

## References

[1] Booth T.H., Nix H.A., Busby J.R., Hutchinson M.F. (2014). BIOCLIM: The first species distribution modelling package, its early applications and relevance to most current MAXENT studies. Divers. Distrib..

[2] Thomas C.D., Cameron A., Green R.E., Bakkenes M., Beaumont L.J., Collingham Y.C., Erasmus B.F.N., De Siqueira M.F., Grainger A., Hannah L. (2004). Extinction risk from climate change. Nature.

[3] Chen I.C., Hill J.K., Ohlemuller R., Roy D.B., Thomas C.D. (2011). Rapid range shifts of species associated with high levels of climate warming. Science.

[4] Pauli H., Gottfried M., Dullinger S., Abdaladze O., Akhalkatsi M., Alonso J.L.B., Coldea G., Dick J., Erschbamer B., Calzado R.F. (2012). Recent Plant Diversity Changes on Europe’s Mountain Summits. Science.

[5] Lee H., Romero J., IPCC, Core Writing Team (2023). Summary for Policymakers. Climate Change 2023: Synthesis Report. Contribution of Working Groups I, II and III to the Sixth Assessment Report of the Intergovernmental Panel on Climate Change.

[6] CMA The Third National Assessment Report on Climate Change was Released; China Meteorological Administration. http://www.cma.gov.cn/2011xwzx/2011xqxxw/2011xqxyw/201412/t20141207_269047.html.

[7] Qin H., Dong G., Zhang Y., Zhang F., Wang M. (2017). Patterns of species and phylogenetic diversity of Pinus tabuliformis forests in the eastern Loess Plateau, China. Forest. Eco. Manag..

[8] Parmesan C., Yohe G. (2003). A globally coherent fingerprint of climate change impacts across natural systems. Nature.

[9] Wake D.B. (2007). Climate change implicated in amphibian and lizard declines. Proc. Natl. Acad. Sci. USA.

[10] Lenoir J., Gegout J.C., Marquet P.A., Ruffray P.D., Brisse H. (2008). A significant upward shift in plant species optimum elevation during the 20th century. Science.

[11] Mckenney D.W., Pedlar J.H., Lawrence K., Campbell K., Hutchinson M.F. (2007). Potential impacts of climate change on the distribution of north american trees. Bioscience.

[12] Kumar P. (2012). Assessment of impact of climate change on rhododendrons in Sikkim himalayas using maxent modelling: Limitations and challenges. Biodivers. Conserv..

[13] Dadashi-Jourdehi A., Shams-Esfandabad B., Ahmadi A., Rezaei H.R., Toranj-Zar H. (2020). Predicting the potential distribution of striped hyena Hyaena hyaena in Iran. Belg. J. Zool..

[14] Jacobson A.P., Gerngross P., Lemeris J.R., Schoonover R.F., Anco C., Breitenmoser-Würsten C., Durant S.M., Farhadinia M.S., Henschel P., Kamler J.F. (2016). Leopard (*Panthera pardus*) status, distribution, and the research efforts across its range. Peerj.

[15] Hayward M.W., Henschel P., O’Brien J., Hofmeyr M., Balme G., Kerley G.I. (2006). Prey preferences of the leopard (*Panthera pardus*). J. Zool..

[16] Athreya V., Odden M., Linnell J.D.C., Krishnaswamy J., Karanth U. (2013). Big cats in our backyards: Persistence of large carnivores in a human dominated landscape in India. PLoS ONE.

[17] Odden M., Athreya V.R., Rattan S.K., Linnell J.D. (2014). Adaptable neighbours: Movement patterns of gps-collared leopards in human dominated landscapes in India. PLoS ONE.

[18] Malhi Y., Doughty C.E., Galetti M., Smith F.A., Svenning J.C., Terborgh J.W. (2016). Megafauna and ecosystem function from the Pleistocene to the Anthropocene. Proc. Natl. Acad. Sci. USA.

[19] Hoeks S., Huijbregts M.A.J., Busana M., Harfoot M.B.J., Jens-Christian S., Santini L. (2020). Mechanistic insights into the role of large carnivores for ecosystem structure and functioning. Ecography.

[20] Ripple W.J., Estes J.A., Beschta R.L., Wilmers C.C., Ritchie E.G., Hebblewhite M., Berger J., Elmhagen B., Letnic M., Nelson M.P. (2014). Status and ecological effects of the world’s largest carnivores. Science.

[21] Sergio F., Caro T., Brown D., Clucas B., Hunter J., Ketchum J., McHugh K., Hiraldo F. (2008). Top predators as conservation tools: Ecological rationale, assumptions, and efficacy. Annu. Rev. Ecol. Evol. Syst..

[22] Ritchie E.G., Johnson C.N. (2009). Predator interactions, mesopredator release and biodiversity conservation. Ecol. Lett..

[23] Estes J.A., Terborgh J., Brashares J.S., Power M.E., Berger J., Bond W.J., Carpenter S.R., Essington T.E., Holt R.D., Jackson J.B.C. (2011). Trophic downgrading of planet earth. Science.

[24] Laguardia A., Kamler J.F., Li S., Zhang C., Zhou Z., Shi K. (2017). The current distribution and status of leopards *Panthera pardus* in China. Oryx.

[25] Jiang Z.G., Wu Y., Liu S.Y., Jiang X.L., Zhou K.Y., Hu H.J. (2021). China’s Red List of Biodiversity· Vertebrates (Vol. I): Mammals (I, II and III).

[26] Kumar B., Babu S., Kumara H.N. (2018). Predicting the potential distribution of the lesser-known endemic madras hedgehog *Paraechinus nudiventris* (order: Eulipotyphla, family: Erinaceidae) in southern india. Mammalia..

[27] Wang S.G., Guo Z.H., Gu B.J., Li T.T., Su Y.B., Ma B.C., Guan H.X., Huang Q.W., Wang F., Zhang Z.J. (2022). Habitat use of the North China leopard (*Panthera pardus japonensis*) in the Liupanshan Mountains and its implications for conservation planning. Biodivers. Sci..

[28] Phillips S.J., Anderson R.P., Schapire R.E. (2006). Maximum entropy modeling of species geographic distributions. Ecol. Model.

[29] Duan Y.Z., Wang H.T., Wang C., Du Z.Y. (2020). Potential distribution of endangered plant *Helianthemum songaricum* in China under climate change. J. Plant. Resour. Environ..

[30] Liu Q., Ye J., Kang Z., Yu G., Yang C., Li J., Tang T. (2025). Reeve’s Muntjac (*Muntiacus reevesi*) Habitat Suitability Under Climate Change Scenarios in Hupingshan National Nature Reserve, China. Animals.

[31] Wang Y.G., Zhang B.R., Zhao R. (2022). Influence of species interaction on species distribution simulation and modeling methods. Chin. J. Appl. Ecol..

[32] Elith J., Graham C.H., Anderson R.P., Dudík M., Ferrier S., Guisan A., Hijmans R.J., Huettmann F., Leathwick J.R., Lehmann A.J.E. (2006). Novel methods improve prediction of species’ distributions from occurrence data. Ecography.

[33] Halvorsen R. (2013). A strict maximum likelihood explanation of MaxEnt, and some implications for distribution modelling. Sommerfeltia.

[34] Ding G.W., Du L.J. (2020). The current status of wild survival of North China leopard. Farmers Consult..

[35] Wang T.J., Li J.P., Tuo J.H. (2022). North China leopard appears in Tuoliang National Nature Reserve, Hebei Province. Land Green..

[36] Han Y.L. (2018). Population quantity investigation of *Panthera pardus fontanierii* of Tieqiao mountain in Shanxi. Shanxi For. Sci. Technol..

[37] Zhang X.F. (2021). Continuous monitoring of *Panthera pardus fontanierii* population quantity in Tieqiao mountain nature reserve of Shanxi Province. Shanxi For. Sci. Technol..

[38] Yang H., Xie B., Zhao G., Gong Y., Mou P., Ge J., Feng L.M. (2021). Elusive cats in our backyards: Persistence of the North Chinese leopard (*Panthera pardus japonensis*) in a human-dominated landscape in central China. Integr. Zool..

[39] Song D.Z., Wang B.P., Jiang J.Y., Wan S.P., Cui S.M., Wang T.M., Feng L.M. (2014). Using camera trap to monitor a North Chinese leopard (*Panthera pardus japonesis*) population and their main ungulate prey. Biodivers. Sci..

[40] Luo W.H., Gao C.Y., Li J.Z., Li C.S., Tang Y.M., Wang J., Jiang G.S., Hua Y. (2020). Spatiotemporal coexistence of North Chinese Leopard (*Panthera pardus japonesis*) and prey in Tie Qiao Shan provincial nature reserve. Acta Ecol. Sinica.

[41] Man H., Huang B.X., Qi J.Z., Jiang G.S. (2022). North Chinese leopard habitat selection in Manghe national natural reserve. Chin. J. Wildl..

[42] Jin T.T., Cao E.J., Gong J. (2022). Spatiotcmporal of variations of vegetation coverage and Its relationships with climate change and human activities in Ziwuling region during 2000–2018. Bull. Soil Water Conserv..

[43] Zhong Z.K., Wang X., Zhang X.Y., Zhang W., Xu Y.D., Ren C.J., Han X.H., Yang G.H. (2019). Edaphic factors but not plant characteristics mainly alter soil microbial properties along a restoration chronosequence of Pinus tabulaeformis stands on Mt. Ziwuling, China. Forest Ecol. Manag..

[44] Ministry of Environmental Protection (2011). National Biodiversity Strategy and Action Plan (2011–2030).

[45] Wang T.M., Feng L.M., Yang H.T., Han B.Y., Zhao Y.H., Li J., Lü X.Y., Zou L., Li T., Xiao W.H. (2016). A science-based approach to guide Amur leopard recovery in China. Biol. Conser..

[46] Liu Y., Huang P., Lin F., Yang W.Y., Gaisberger H., Christopher K., Zheng Y.Q. (2019). MaxEnt modelling for predicting the potential distribution of a near threatened rosewood species (*Dalbergia cultrata* Graham ex Benth). Ecol. Eng..

[47] Meinshausen M., Nicholls Z.R.J., Lewis J., Gidden M.J., Wang R.H.J. (2020). The shared socio-economic pathway (SSP) greenhouse gas concentrations and their extensions to 2500. Geosci. Model Dev..

[48] Ismaili R.R.R., Peng X., Li Y., Ali A., Ahmad T., Rahman A.U., Ahmad S., Shi K. (2024). Modeling Habitat Suitability of Snow Leopards in Yanchiwan National Reserve, China. Animals.

[49] Xiao C.L., Qian C., Huang A.N., Guo R.X., Kuang X.Y. (2023). Evaluation of AMIP models from CMIP6 in simulating winter surface air temperature trends over Eurasia during 1998–2012 based on dynamical adjustment. Clim. Dynam..

[50] Li Q., Qi Y., Wang Q., Wang D.L. (2022). Prediction of the Potential Distribution of *Vaccinium uliginosum* in China Based on the Maxent Niche Model. Horticulturae.

[51] Roshani, Rahaman M.H., Masroor M., Sajjad H., Saha T.K. (2024). Assessment of habitat suitability and potential corridors for Bengal Tiger (*Panthera tigris tigris*) in Valmiki Tiger Reserve, India, using MaxEnt model and Least-Cost modeling approach. Environ. Model. Assess..

[52] Hou J.L., Xiang J.G., Li D.L., Liu X.H. (2023). Prediction of potential suitable distribution areas of Quasipaa spinosa in China based on MaxEnt optimization model. Biology.

[53] Li X., Huang Y.Y., Ruan T., Wei W. (2022). Maxent model-based evaluation of habitat suitability of Chinese red panda in Qionglai mountains. J. Guizhou Norm. Univ. Nat. Sci..

[54] Hill M.P., Hoffmann A.A., McColl S.A., Umina P.A. (2012). Distribution of cryptic blue oat mite species in Australia: Current and future climate conditions. Agric. For. Entomol..

[55] Wang R.L., Li Q., Feng C.H., Shi Z.P. (2017). Predicting potential ecological distribution of Locusta migratoria tibetensis in China using MaxEnt ecological niche modeling. Acta Ecol. Sin..

[56] Xu D.P., Zhou Z.H., Wang R.L., Ye M., Pu B. (2019). Modeling the distribution of Zanthoxylum armatum in China with MaxEnt modeling. Glob. Ecol. Conserv..

[57] Phillips S.J., Dudík M. (2008). Modeling of species distributions with Maxent: New extensions and a comprehensive evaluation. Ecography.

[58] Jackson C.R., Robertson M.P. (2011). Predicting the potential distribution of an endangered cryptic subterranean mammal from few occurrence records. J. Nat. Conserv..

[59] Clark J.T., Fei S., Liang L., Rieske L.K. (2012). Mapping eastern hemlock: Comparing classification techniques to evaluate susceptibility of a fragmented and valued resource to an exotic invader, the hemlock woolly adelgid. For. Ecol. Manag..

[60] Majumder A., Suryan T., Rizvi T., Tripathi R.M., Nag S. (2024). Assessment of the population and suitable habitat for a leopard (*Panthera pardus*) in the urban landscapes of Central India. Eur. J. Wildl. Res..

[61] Merow C., Smith M.J., Silander J.A. (2013). A practical guide to MaxEnt for modeling species’ distributions: What it does, and why inputs and settings matter. Ecography.

[62] Fourcade Y., Besnard A.G., Secondi J. (2018). Paintings predict the distribution of species, or the challenge of selecting environmental predictors and evaluation statistics. Glob. Ecol. Biogeogr..

[63] Nolan C., Overpeck J.T., Allen J.R.M., Anderson P.M., Betancourt J.L., Binney H.A., Brewer S., Bush M.B., Chase B.M., Cheddadi R. (2018). Past and future global transformation of terrestrial ecosystems under climate change. Science.

[64] Tape K.D., Christie K., Carroll G., O’Donnell J.A. (2016). Novel wildlife in the Arctic:the influence of changing riparian ecosystems and shrub habitat expansion on snowshoe hares. Glob. Change Biol..

[65] Allen M.R., Barros V.R., Broome J., CHRIST R., Church J., Clarke L., Cramer W., Dasgupta P., DUBASH N., Edenhofer O. (2014). Climate Change 2014: Synthesis Report. Contribution of Working Groups I, II and III to the Fifth Assessment Report of the Intergovernmental Panel on Climate Change.

[66] Stirling I., Derocher A.E. (2012). Effects of climate warming on polar bears: A review of the evidence. Glob. Change Biol..

[67] Lang P.F. (2020). Study on the Distribution and Changes of Leopards (*Panthera pardus*) in China in the Past 300 Years.

[68] Gethöffer F., Keuling O., Maistrelli C., Ludwig T., Siebert U. (2023). Heavy Youngsters-Habitat and climate factors lead to a significant increase in body weight of wild boar females. Animals.

